# O.5.1-2 Associations of active school transport and leisure-time physical activity with educational and school-related mental health outcomes among Finnish adolescents: a nationwide cross-sectional study

**DOI:** 10.1093/eurpub/ckad133.232

**Published:** 2023-09-11

**Authors:** Juuso J Jussila, Anna Pulakka, Jaana I Halonen, Paula Salo, Sara Allaouat, Santtu Mikkonen, Timo Lanki

**Affiliations:** Institute of Public Health and Clinical Nutrition, University of Eastern Finland, Kuopio, Finland; Department of Health Security, Finnish Institute for Health and Welfare (THL), Helsinki, Finland; Research Unit of Population Health, Faculty of Medicine, University of Oulu, Oulu, Finland; Population Health Unit, Finnish Institute for Health and Welfare (THL), Helsinki, Finland; Department of Health Security, Finnish Institute for Health and Welfare (THL), Helsinki, Finland; Department of Psychology and Speech-Language Pathology, University of Turku, Turku, Finland; Institute of Public Health and Clinical Nutrition, University of Eastern Finland, Kuopio, Finland; Department of Health Security, Finnish Institute for Health and Welfare (THL), Helsinki, Finland; Department of Technical Physics, University of Eastern Finland, Kuopio, Finland; Department of Environmental and Biological Sciences, University of Eastern Finland, Kuopio, Finland; juuso.jussila@uef.fi; Institute of Public Health and Clinical Nutrition, University of Eastern Finland, Kuopio, Finland; Department of Health Security, Finnish Institute for Health and Welfare (THL), Helsinki, Finland; Department of Environmental and Biological Sciences, University of Eastern Finland, Kuopio, Finland; juuso.jussila@uef.fi

## Abstract

**Purpose:**

Physically active pupils may be better and more resilient learners. However, it is unknown whether active school transport (walking or cycling to school) could affect educational and school-related mental health outcomes. We examined the associations of active school transport and leisure-time moderate-to-vigorous physical activity with perceived academic performance, self-reported competency in academic skills (concentration in classroom, writing, reading, and calculation), school burnout, and school enjoyment among Finnish adolescents.

**Methods:**

We included 34103 eighth and ninth graders (mean age 15.4 years; 53% girls) from the nationwide School Health Promotion study cohort of 2015. We used logistic regression, adjusting for major sociodemographic, lifestyle, and physical activity covariates, to estimate the associations.

**Results:**

We observed small but positive associations between active school transport and educational outcomes. For example, compared with non-active transport, 10-30 minutes of active school transport a day was associated with 30% higher odds of high perceived academic performance (OR 1.30, 95% CI 1.21-1.40) and 16% higher odds of high competency in reading (OR 1.16, 95% CI 1.07-1.26). Any dose of active school transport was also associated with higher school enjoyment, but no relationship with school burnout was observed. Compared with inactivity, higher doses of leisure-time physical activity were robustly associated with better educational outcomes. For example, the most physically active adolescents had 85% higher odds of high perceived academic performance (OR 1.85, 95% CI 1.65-2.08). Regarding competency in academic skills, the strongest association was observed for calculation (OR 1.57, 95% CI 1.39-1.77). Engaging in leisure-time physical activity, at any dose, was also associated with lower odds of school burnout, and higher odds of school enjoyment.

**Conclusions:**

Compared with active school transport, engaging in leisure-time moderate-to-vigorous physical activity seems to be more strongly associated with high perceived academic performance, high competency in academic skills, and less school burnout among youth. Despite this, walking and cycling to school might lead to minor improvements in classroom performance and school enjoyment.

**Funding Source:**

Juuso J. Jussila was supported by the Academy of Finland, Strategic Research Council (#336003).

